# Adaptation of a Cervical Cancer Education Program for African Americans in the Faith-Based Community, Atlanta, Georgia, 2012

**DOI:** 10.5888/pcd11.130271

**Published:** 2014-04-24

**Authors:** Venice Haynes, Cam Escoffery, Corinthia Wilkerson, Rashida Bell, Lisa Flowers

**Affiliations:** Author Affiliations: Cam Escoffery, Emory University, Rollins School of Public Health, Atlanta, Georgia; Corinthia Wilkerson, Spelman College, Atlanta, Georgia; Rashida Bell, Spirit Foundation Inc, Lithonia, Georgia; Lisa Flowers, Emory University School of Medicine, Atlanta, Georgia.

## Abstract

**Background:**

From 1999 through 2009, African American women in the United States had the second highest incidence rates of cervical cancer and were more likely to die from cervical cancer than women of other races. *Con Amor Aprendemos* (CAA) is an intervention created to educate the Latino community to reduce their risk for cervical cancer and diseases related to human papilloma virus (HPV). CAA was adapted to With Love We Learn (WLWL) to prevent cervical cancer and HPV in African American communities.

**Community Context:**

Health ministries of 2 churches in the Atlanta area partnered with the Spirit Foundation Inc to adapt CAA to WLWL by tailoring the curriculum to the African American faith-based community.

**Methods:**

The National Cancer Institute’s Research to Reality (R2R) mentorship program pair collaborated with program staff on an adaptation summary form, a tool to document and assist with adapting the program curriculum with fidelity. Trainers, faith leaders, and participants adapted the program in 4 phases: 1) review of the CAA curriculum, 2) a focus group discussion to determine changes for the WLWL curriculum, 3) train-the-trainer sessions on program delivery, and 4) a pilot intervention and follow-up focus group to evaluate the new curriculum.

**Outcomes:**

The CAA/WLWL curriculum was adapted and piloted in a faith-based setting. Adaptations to the CAA program included pictures, games, statistics on cervical cancer, dialogues, and delivery of curriculum.

**Interpretation:**

Community engagement in the adaptation of WLWL through various methods was critical to tailoring an evidence-based program to a new population and setting.

## Background

An estimated 12,340 cases of invasive cervical cancer are expected to be diagnosed and 4,030 deaths from cervical cancer are expected for 2013 (1). Cervical cancer is the second most common cancer among women worldwide, and minorities experience disparities in cervical cancer incidence and mortality rates. From 1999 through 2009, African American women in the United States had the second highest incidence rates of cervical cancer, yet they were more likely to die from cervical cancer than women of other races (2).

Many intervention strategies have been studied to increase cervical cancer screening. *The* *Guide to Community Preventive Services* recommends client reminders, small media (eg, printed materials), one-on-one education, provider assessment and feedback, and provider reminders as strategies to increase cervical cancer screening (3). However, only 3 of 8 evidence-based interventions (EBIs) that focused on African American women were found on the National Cancer Institute’s Research-Tested Intervention Programs website (4). Therefore, because of limitations in evidence-based options, community organizations may adapt EBIs for a new community or audience. Adaptation is defined as the degree to which an EBI is changed or modified during adoption and implementation to suit the needs of the setting or to improve the fit to local conditions (5). Several models exist that provide considerations and processes for program adaptation and implementation (6–8). In making program adaptations, it is important to address cultural mismatches and follow processes to ensure program changes fit the local populations or conditions (9).

In response to the high prevalence of cervical cancer and human papilloma virus (HPV) among Latino women, the Spirit Foundation Inc, in partnership with the American Cancer Society, South Atlantic Division, created the *Con Amor Aprendemos* (CAA) program for Latino couples to increase knowledge about risk and behaviors leading to cervical cancer and HPV-related diseases. This 7-week intervention is implemented by trained *promotoras*, or community health workers, in faith-based and community organizations. Sessions last an average of 2 hours, and each session follows a specified curriculum covering topics on anatomy, sexually transmitted infections (STIs), cervical cancer and HPV, dialogues, role playing, presentation of skits to community or church members, and education about the HPV vaccine. An extensive 2-day train-the-trainer course is a vital component of the CAA program to train a team of community members in health ministries on how to deliver the curriculum to the faith-based community. Couples are targeted to participate in the program in efforts to reduce the anxiety of the male–female partner dialogue regarding HPV and risks associated with contracting cervical cancer. The program uses innovative tools, such as a “ring of knowledge” — where program participants collect small notecards with information throughout the course of the program — games to build awareness about STIs, anatomy labeling, and a “parking lot” for participants to ask personal questions anonymously.

CAA has been implemented in Latino communities in Georgia, Nicaragua, El Salvador, and Bolivia. This intervention has been piloted in El Salvador with preliminary results demonstrating increased knowledge about HPV and Cervical Cancer (10). This article documents the process of adapting CAA for the African American community and highlights the data collection methods, adaptation of program information, and the roles of health ministry leaders to make suggestions about program modifications for a new population. The adapted program is called *With Love We Learn* (WLWL).

## Community Context

After successful implementation in multiple Latino communities, the Spirit Foundation Inc. considered it essential to replicate the program in the African American community in light of the high incidence of cervical cancer among African American women. Without appropriate education reinforcing the importance of screening and follow-up, the rates of cervical cancer will continue to rise among African American women. As it was in the Latino community, a faith-based setting was chosen for adaptation of CAA in the African American community because of the strong historical ties the African American community has with the church. Faith-based organizations have become one of the most common vehicles for the dissemination of prevention efforts and addressing health concerns for that population (11,12).

In October 2011, the Spirit Foundation partnered with 2 churches in the metro Atlanta area that would adopt WLWL in their health ministries. These churches have predominantly African American populations with congregations of 4,000 members or more (Table 1). Health ministry leaders from these churches were invited to participate in the community assessment and adaptation discussions.

**Table 1:** Profile of Churches Participating in Adaptation of *Con Amor Aprendemos* to With Love We Learn, Atlanta, Georgia, 2012

Characteristic	Church 1: Baptist	Church 2: Nondenominational
Primary race/ethnicity	African American	Predominantly African American
Location/neighborhood	Decatur, Georgia	Decatur, Georgia
Size of congregation	4,000+	7,000+
Health ministry present?	Yes	Yes
Participants in train-the-trainer workshop	4	2

## Methods

**Figure 1:**
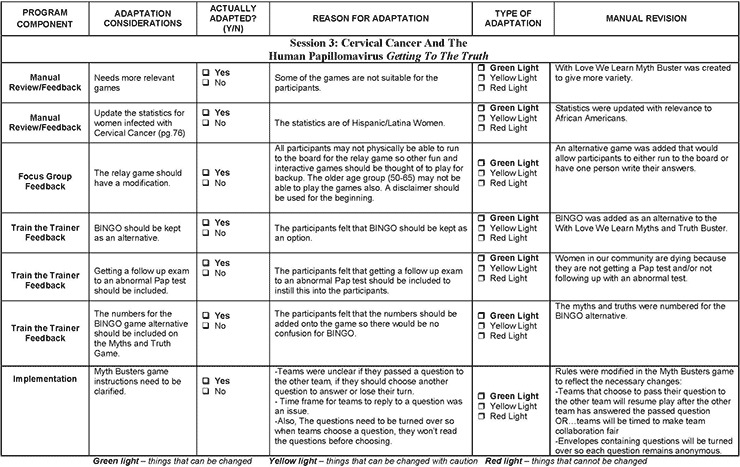
Example of Summary Form Used During Adaptation of *Con Amor Aprendemos* to With Love We Learn Programs to Educate Communities About Cervical Cancer and HPV-Related Diseases, Atlanta, Georgia, 2012.

In 2011, the National Cancer Institute piloted the Research to Reality (R2R) mentorship program as a capacity-building initiative for public health practitioners to gain hands-on experience in evidence-based programs and decision-making practices. Mentees were paired with seasoned cancer control practitioners and worked on a year-long cancer control and prevention project to learn and apply new skills in evidence-based public health practices. The project for the Georgia R2R pair focused on the adaptation of CAA to WLWL. Orientation meetings were held with program developers and staff members to learn the details of the program, and worked for several months to gather information that would inform the key phases at which data would be gathered in the adaptation process. The adaptation summary form was the primary tool used among project staff to document the changes at each phase (Figure 1). The program was adapted in 4 phases: 1) review of the CAA curriculum, 2) a focus group discussion to determine changes for the WLWL curriculum, 3) train-the-trainer sessions on program delivery, and 4) a pilot intervention and follow-up focus group to evaluate the new curriculum.

**Table:** 

Figure 1 illustrates the summary form used throughout the adaptation phase that captures the changes that were made at each stage.
Across the top of the table are categories that begin with program component, at what stage in the adaptation took place, adaptation considerations, were the considerations actually adapted, the reason for adaptation, the type of adaptation (red, yellow or green light), and what revisions were made to the manual.
The figure is specific to Session 3: "Cervical Cancer and the Human Papillomavirus" curriculum because session 3 had recommendations that touched all 4 phases or program components: manual review/feedback, focus group feedback, train-the-trainer feedback, and implementation. These phases are along the first column of the table under program component.
Rows 1 and 2, the adaptation considerations during the manual review/feedback phase, the need for more relevant games and to update statistics for women with cervical cancer. These changes were adapted because some of the games were not suitable for the participants, and the statistics were for Hispanic/Latina women. This was considered a green light adaptation because it did not compromise the fidelity of the program’s curriculum. A green light adaptation indicates components that can be changed, a yellow light adaptation indicates components that can be changed with caution, and a red light adaptation indicates components that cannot be changed. The manual revision included the Myth Buster game that was created to give more variety, and the statistics were updated with relevance to African Americans.
For row 3, the focus group feedback adaptation consideration was to modify the Relay Game. This suggestion was adapted because all participants may not physically be able to run to the board for the game. The older age group (50-65 y) may not be able to play the games, and a disclaimer should be made as well. This was also a green light adaptation. The manual revision was that 1 participant could run to the board or that 1 person would write down the answers.
Rows 4, 5, and 6 discuss feedback from the train-the-trainer program component. The adaptation considerations at this phase included keeping BINGO as an option, and including language about obtaining a follow-up exam after an abnormal pap. These recommendations were made and adapted because BINGO was considered a good alternative to other games and because women are dying because they are not following up after an abnormal Pap test.
Row 7 provides considerations for clarifying the Myth Busters game for logistics on how the game should be played.

Six health ministry leaders from the 2 partner churches reviewed the CAA program manual for general understanding of the overall curriculum. Reviews were conducted over a 2-week period, and leaders were provided with a deadline by the program developers to provide their initial feedback. Detailed notes were written throughout the manual on specific areas suggested for revision for the African American audience.

Following the manual review, 6 health ministry leaders met at one of the partner churches for a 2-hour focus group. During the focus group, leaders and program developers discussed the detailed components of the curriculum and explained how each session was to be conducted. Participants talked through the notes written throughout the manual on sections that needed revisions. A group of stakeholders from academia, government, and community collaborated on developing the focus group guide to gather detailed information about key changes to the curriculum. There were specific inquiries were made about changes to the core content and pedagogical and implementation components (13). The guide had 4 major sections that addressed the overall program and delivery, program incentives, program materials, and technical assistance in delivering the program (Appendix A). The focus group discussion was audio taped and transcribed verbatim. NVivo 10 software (QSR International, Burlington, Massachusetts) was used for data storage, retrieval, and analysis. A content analysis was performed to identify the range of responses and major themes related to revisions to the WLWL manual (14) (Table 2). A summary of the manual changes by session was also recorded on the adaptation summary form (Figure 1.)

**Table 2:** Focus Group Comments from Trainers Related to Adapting *Con Amor Aprendemos* to With Love We Learn, Atlanta, Georgia, 2012

Topic	Trainer Comment
**Overall Program and Delivery**	
Age categories appropriate	“. . . you may want 3. My guess, and I just suggest 21 to 29, 3o to 44, 45 to 60 or 45 and above.”
	“I would probably do it like he did, like you have the younger adults 21 to 29, then say your middle-aged group from 30 to 44, then maybe 45 to 65, and I think the groups will have more in common that way.”
Who should participate	“. . . many of the people could use this most may not actually be in couples situations.”
	“I think there is a distinction. One is not more valid than the other. But if this is specifically to enrich the understanding and interaction between couples then the way the information is versed and presented has to reflect that and show the ways that it strengthens what’s going on with a couple as it pertains to the information that you’re giving versus just it being sex education.”
**Program incentives**	
Motivation for keeping couples in the program	“I mean for the couples they always want a weekend getaway or something.”
**Materials/intervention**	
Thoughts about games in the manual	“I really like the idea of having the games.”
	“I was going to say have an alternative game for that same time in the class, so based on your class, especially if it’s the younger group who typically are a little — can’t sit as long or don’t have the tolerance to actually wait for somebody to get Bingo. You might get a game that’s a little faster moving or if you have a class and you know that your class is like that. You have an option to Bingo. Maybe you can choose based on kind of your class.”
Session 2 (Pictures associated with STIs and HPV)	“That's too bold”; “That's reality”; “Yeah, leave it.”
Session 5 (Act It Out)	“Yeah, dialoging and doing the props and the — I think — we like to do miming and — so I think acting it out — most people like to participate in being somebody they aren't.”
Session 6 (community presentations)	“I think that goes into the campaign, the drama presentation, the skits, the video — everything that we kind of laid out in terms of having the Pastors to get on board and involve the leadership. Pretty much those things, they go into that. Those are the best ways.” “I think we really got to push out the fun aspects. This is going to be a fun environment. You're not coming here to sit here and be like bored to death. It's fun.” Abbreviations: STIs, sexually transmitted infections; HPV, human papilloma virus.

After incorporation of the recommended changes, the WLWL manual was revised and a 2-day training workshop was conducted to train the 6 health ministry leaders on the revised curriculum. The objective of the train-the-trainer program for CAA/WLWL is to establish community educators who are motivated to address the community with accurate knowledge of HPV and cervical cancer. The training sessions were conducted on location to provide a familiar environment for the trainers. Refresher sessions were provided closer to program implementation to ensure that trainers remained comfortable teaching information accurately and did not deviate from the set topics of the curriculum.

The first pilot of WLWL in the churches began in May 2013 with 15 (13 female and 2 male) participants. Participants were recruited through church announcements, fliers, and word of mouth from the health ministry leaders. Not all of the participants were couples, however all 15 participants completed the adapted program in June 2013. On completion, participants and trainers were invited to participate in a focus group discussion to gather their feedback. Participants were asked their opinions about the marketing of the program to their congregation, their comfort level with questionnaires that asked about sensitive and personal information, receptivity to the session topics and components, and their recommendations for recruiting participants in future programs. Data gathered from the pilot group of WLWL participants served as the final data collection point to include on the adaptation summary form for the last manual revisions.

The overall program curriculum and the focus group guide was submitted under the Emory University Social and Behavioral Institutional Review Board and received exemption from full review. Trainers were compensated for their time for the manual review, focus group discussion, and train-the-trainer session. and participants in the pilot program were compensated for their feedback during the focus group. The R2R mentorship pair, program developers, and WLWL project staff followed adaptation guidance from the literature (15) to summarize all of the suggestions provided for tailoring, ensuring that the recommended changes did not affect the fidelity to the original core elements or required elements of the program (16).

## Outcomes

**Figure 2:**
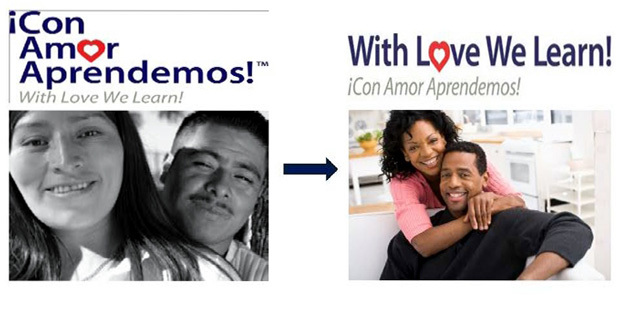
Changes to *Con Amor Aprendemos* for the Cover of With Love We Learn Manual, Atlanta, Georgia, 2012. [A text description of this figure is also available.]

Overall, the program was well received by church leaders, trainers, and participants of the pilot intervention. Each phase of the adaptation process included valuable recommendations for the WLWL curriculum (Figure 2). Suggestions for content tailoring included changes to the cover, games related to session topics, pictures to reflect African Americans, and more effective dialogue to deliver the content of the sessions to an African American population. The modification of relevant health statistics addressing screening rates and cervical cancer mortality for African American women was also critical to effectively communicate risk data to participants. Other modifications included recommendations for stratifying the program by age groups to promote easier conversations with peers closer in age. The frequency of the sessions was condensed to twice a week for 3 weeks to retain members’ participation throughout the program (Table 3). Core elements of the program, including the session topics and the use of games, pictures, posters, and dialogues for learning the material, were not changed. Although these materials were welcomed and reported to be helpful in learning, suggestions were given to make them relevant to both younger and older African American audiences in the community. The program developer, a gynecologist, and other program staff reviewed the comments and modified the pilot WLWL curriculum.

**Table 3:** Examples of Changes From *Con Amor Aprendemos* to With Love We Learn*,* Atlanta, Georgia, 2012

Manual Component	*Con Amor Aprendemos*	Comment/Reason for Adaptation	*With Love We Learn*
Pictures	See the first picture in Figure 2	The photos of an African American couple relate to the African American community more than the Hispanic/Latino couple.	See the second picture in Figure 2
Sessions	7 Sessions – 1 session/week	The participants would be taking time out of their busy schedules; be mindful of the session days and lengths.	6 sessions — 2 sessions/week for 3 weeks
Titles of health leaders	*Promotoras*	The English translation is more suitable for the African American community.	Health educators
Games	Games (Bingo) to learn about STI’s in the Hispanic community	The younger crowd would like a more fun and interactive game.	With Love We Learn “Myth Buster” was created to give more variety.
Statistics	Statistics for cervical cancer among Latina women	Update the statistics for women infected with cervical cancer.	Statistics were updated with relevant data on cervical cancer for African American women.
Dialogues/role playing	Settings for dialogues should be culturally relevant (ie, salon restaurant)	The settings for the dialogues need to be more relevant to the African American community.	Some of the scripts for the dialogues were edited to make them more relevant to the African American community.
Dialogues/role playing	Conversation and role playing between Hispanic males	Many black men do not like to talk, so it will be important to create leading questions instead of open-ended questions to stimulate conversation and sharing.	The language in the dialogues (particularly the one between the two men) was edited in a way that would make them feel more comfortable discussing the topic.

## Interpretation

Program adaptation is an important process in the translation of evidence-based programs into practice. A systematic process for adaptation is necessary to learn what potential changes can be made to a program to ensure its suitability for another population and also balance fidelity to the original program. Community engagement is equally important for this adaptation process to garner insights from key constituents and understand contextual issues in matching interventions to particular communities. Castro and colleagues summarize key processes in program adaptation including assessing your audience, selecting an evidence-based approach, preparing for adaptation with focus groups or topical expert review, adapting the program, testing adaptation materials, and refining adaptation (15,17). We followed the guidance for adaptation preparation with multiple data collection methods to learn about the target audience (needs assessment) and assist in making decisions on what program components to change or refine.

One of the most important considerations for adapting community programs is to understand both the intervention and the components required for program fidelity to remain uncompromised. Additionally, it is equally important to take the time to foster the right partnerships with faith-based institutions and involve the right stakeholders’ perspectives to ensure the programs’ success. Substantial organization and investment of time are needed to collect data at multiple points. Working closely with the program developers and staff on organizing the data collection throughout the adaptation phases was extremely helpful in keeping the project on track and without losing the fidelity of the original program. Having a resource like the adaptation summary form referenced throughout this case study, is highly recommended to keep records of the changes in a multiphase process.

The data collection and subsequent adaptation of the CAA program involved a variety of stakeholders from program developers to key members of the faith-based organizations to identify areas of original program mismatches (eg, race, community context) to the African American population. The multiple types of data collection generated many recommendations for program changes to increase the fit of the cervical cancer prevention program to African American church members. Many of our program changes fit into the area of context modification (eg, setting, population) and content modification (eg, tailoring, substituting elements) found in a recent review of adaptation of evidence-based interventions (18).

We involved the program developers in this adaptation process in order to speak to the dynamics of the program, the role of the faith-based organization that would be implementing it, and to make informed decisions on what to change in the program. This strategy has been suggested as a way to ensure that the modifications are justifiable and that changes to the content, duration, or delivery style of the program will not diminish the program’s effectiveness (19,20). Linking program developers to community organizations that are adopting evidence-based programs can increase translations of EBIs in that developers can address issues related to program implementation and adaptation (21). Decisions were made by the developers to change certain CAA content and implementation recommendations if they were not deemed to be “red light” adaptations (eg, theory change, dose, elimination of core elements) that would potentially jeopardize program fidelity (13).

Dissemination of evidence-based practices is increasing in the community. Many community organizations are attempting to adapt packaged programs for their own populations and settings. This project has illustrated a method for sequential data collection to inform the adaptation process. Future research should further explore how community-based participatory processes with key community members and organizations can inform program adaptations of evidence-based interventions and testing of adapted programs in communities for effectiveness.

## Appendix A. Focus Group Guide Questions for With Love We Learn Trainers, by Topic, Atlanta, Georgia, 2012

**Table:**

Topic	Questions
Program and delivery	Generally couples of different ages are combined together when this program is given to the community. However we have divided the couples based on 2 defined age groups. The 2 age groups are 21-29 and 30-65. Are these categories appropriate to allow for open conversation? In addition, we have defined the program population to be individuals who are married, engaged, or in long-term relationships. What is your opinion about who should participate in the program? Thinking about your members, how would they feel about a program to promote women’s health and getting a pap test? How will your members react to a program which addresses sensitive issues such as: male and female anatomy sexually transmitted infections monogamy condoms different forms of sexual activities vaccines What are your thoughts about using the church to deliver health messages or materials? Would you change anything related to the delivery of the program? If so, what would you change? What issues and challenges do you foresee in churches delivering health interventions such as this one? Do you have any advice about delivering a health program in a church setting? If yes, please tell us more.
Incentives	What would motivate your members and couples to participate in this program? What strategies or incentives would you recommend to recruit them into the program? What about for keeping them in the program?
Materials/curriculum	What changes would you make to the materials such as information or pictures that would make the program work for your members? What are your thoughts about the games in the manual? What are your suggestions for games to be used to reinforce the information in the sessions? Anatomy game STI game Bingo: Truth and myth Are the symbols used in the manual understandable? Are the educational tools and resources easy to use (STI chart and media material)? Chart Female anatomy coloring sheets Rings of knowledge Parking Lot Concentration game Dialogues Do you think the pre- and posttests are clear and understandable for the population in your church? Sessions 1 through 6: were the materials understandable and clear?
Technical assistance	We will have training on how to deliver the intervention. What other things could help the faith-based leaders with conducting the program? If booster sessions or follow-up training are needed, what is the best way to offer these trainings?

Abbreviation: STI, sexually transmitted infection.
